# Expansion of ruminant-specific microRNAs shapes target gene expression divergence between ruminant and non-ruminant species

**DOI:** 10.1186/1471-2164-14-609

**Published:** 2013-09-10

**Authors:** Hua Bao, Arun Kommadath, Xu Sun, Yan Meng, Adriano S Arantes, Graham S Plastow, Le Luo Guan, Paul Stothard

**Affiliations:** 1 Department of Agricultural, Food and Nutritional Science, University of Alberta, Edmonton, Alberta, Canada

## Abstract

**Background:**

Understanding how species-specific microRNAs (miRNAs) contribute to species-specific phenotypes is a central topic in biology. This study aimed to elucidate the role of ruminant-specific miRNAs in shaping mRNA expression divergence between ruminant and non-ruminant species.

**Results:**

We analyzed miRNA and mRNA transcriptomes generated by Illumina sequencing from whole blood samples of cattle and a closely related non-ruminant species, pig. We found evidence of expansion of cattle-specific miRNAs by analyzing miRNA conservation among 57 vertebrate species. The emergence of cattle-specific miRNAs was accompanied by accelerated sequence evolution at their target sites. Further, the target genes of cattle-specific miRNAs show markedly reduced expression compared to their pig and human orthologues. We found that target genes with conserved or non-conserved target sites of cattle-specific miRNAs exhibit reduced expression. One of the significantly enriched KEGG pathway terms for the target genes of the cattle-specific miRNAs is the insulin signalling pathway, raising the possibility that some of these miRNAs may modulate insulin resistance in ruminants.

**Conclusions:**

We provide evidence of rapid miRNA-mediated regulatory evolution in the ruminant lineage. Cattle-specific miRNAs play an important role in shaping gene expression divergence between ruminant and non-ruminant species, by influencing the expression of targets genes through both conserved and cattle-specific target sites.

## Background

It has long been hypothesized that differences in gene expression contribute extensively to phenotypic differences among species [1,2]. Numerous studies have investigated the effects of cis-acting elements and trans-acting proteins on gene expression divergence [3-5]. A recently discovered class of regulatory RNA molecules called miRNAs is known to play an important role in gene expression. It is now predicted that nearly 50% of mammalian mRNAs are regulated at the translational level by miRNAs [6,7]. Many miRNAs exhibiting both broad sequence and expression conservation among animal species have been identified [8,9]. However, recent high-throughput small RNA sequencing and comparative genomic studies have led to the discovery of a large number of miRNAs with limited species conservation [10-17]. Gene expression regulation by these miRNAs, some of which may be species-specific, may be one of the important mechanisms behind some of the expression and phenotype divergence observed among species.

In this study, we aimed to investigate how species-specific miRNAs drive gene expression divergence by identifying cattle-specific miRNAs and characterizing their contribution to cattle-specific gene expression divergence using Illumina sequencing and comparative genomics. Dramatic physiological and phenotypic differences exist between ruminant and non-ruminant mammalian species. For example, volatile fatty acids produced as by-products of the microbial fermentation in the rumen are used as the major source of energy in ruminants as opposed to glucose absorbed from the small intestine in non-ruminants. Because of this difference in nutrient usage, ruminants are less sensitive to insulin than non-ruminants [18]. Several major genes involved in the insulin pathway, including INSR, GLUT1, GLUT4 and PI3K, show decreased expression in ruminants compared to non-ruminants [19-21].

## Results

### Profiling of miRNAs from the whole blood of cattle and pigs

To assess miRNA repertoire divergence between a ruminant and a closely related non-ruminant species, we sequenced miRNAs isolated from whole blood samples of cattle and pigs. From three blood samples of each species, an average of 26 million reads (per sample) (Additional file 1) were obtained, and approximately 89% of the reads could be mapped to the corresponding reference genome assemblies: UMD3.1 for cattle and Sscrofa10.2 for pigs. A total of 676 and 257 miRNAs have been reported for cattle and pigs respectively in miRBase release 18. Using the miRNA discovery software, miRDeep2 [22], we identified at total of 228 and 148 known (previously reported in miRBase) miRNAs expressed in the blood of cattle and pig respectively (Table 1). The miRDeep2 software can also predict novel miRNAs using a probabilistic model of miRNA biogenesis to score compatibility of the position and frequency of sequenced RNA with the secondary structure of the miRNA precursor. The *de novo* prediction strategy employed by miRDeep2 was able to identify 87% and 90% of the miRNAs observed in cattle and pig respectively through mapping to known miRNAs in miRBase. It was estimated that a miRDeep2 score cutoff of 5 corresponds to a true positive prediction percentage greater than 90%, and a signal-to-noise ratio > 10, criteria that the authors of the software recommend [22]. With these criteria, we predicted 33 and 54 novel miRNAs in cattle and pig respectively (Table 1). Thus a substantially higher number of miRNA species were identified in cattle blood compared to pig blood, and several of the miRNAs identified in this work have not been previously described. The number of reads detected for each of the known miRNAs is summarized in Additional file 2 and the sequences and genomic positions of novel miRNAs are shown in Additional file 3.

**Table 1:** Numbers of miRNAs identified from cattle and pigs

**Sample ID**	**Known**	**Novel**	**Total**
**Cattle**			
bta-1064	245	48	293
bta-1146	258	53	311
bta-1151	250	51	301
All miRNAs^*^	228	33	261
**Pig**			
ssc-404 F	169	94	263
ssc-406 F	166	112	278
ssc-422 F	153	70	223
All miRNAs^*^	148	54	202

^*^ Refers to miRNAs with greater than 5 reads in all 3 samples.

### Expansion and diversification of cattle-specific miRNAs

Since a substantially higher number of expressed miRNAs were identified in cattle relative to pigs, we wondered if cattle possess additional miRNA genes not found in non-ruminant genomes. To investigate this possibility, we analyzed the conservation of cattle miRNAs across 57 vertebrate species with good quality genome assemblies available in the Ensembl database, version 71 (http://www.ensembl.org/index.html). Our analyses revealed an overall expansion of miRNA repertoire in cattle. About 20% (46 of 228) of the known and over 76% (25 of 33) of the novel cattle miRNAs did not have homologs in any of the 57 vertebrate species (Table 2). We refer to these 71 miRNAs as ‘cattle-specific’ (read counts provided in Additional file 4). The miR-2284 family with 24 members reported in miRBase 18 is the largest miRNA family in cattle. Based on our conservation analysis, this family is also cattle-specific.

**Table 2:** Conservation of cattle miRNAs across vertebrate species

**Category**	**Known**	**Novel**	**All**
Cattle-specific miRNAs	46	25	71
Non-cattle-specific miRNAs	182	8	190

**Figure 1:**
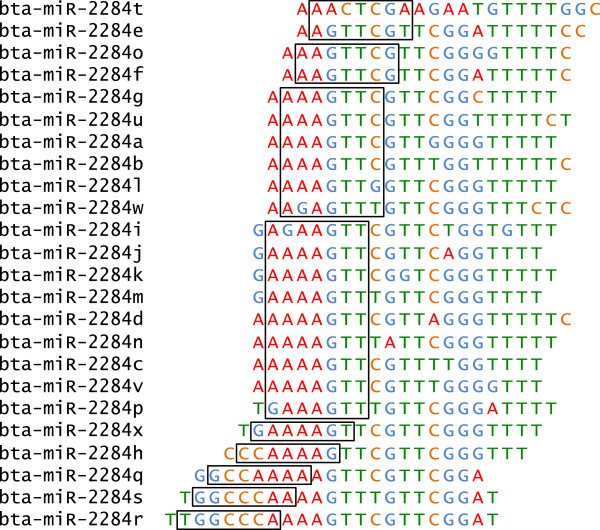
**Evolution of the miR-2284 family by seed shifting and point mutation.** Alignment of the mature cattle miRNA sequences in the mir-2284 family shows the presence of sequence substitutions and differences in the position of the seed region (positions 2–8, boxed).

After a new miRNA gene has emerged, through duplication of an existing gene for example, it can be further diversified in different ways. A seed region of about 7 nucleotides in length at the 5' end of an animal miRNA is thought to be an important determinant of target specificity [23]. One of the mechanisms for acquiring divergent function is seed shifting, wherein the dominant mature miRNA isoform is shifted by one to several nucleotides in the 5’ or 3’ direction relative to its original position [8,14,24]. The miR-2284 family with 24 members provides us with an opportunity to investigate how different mechanisms shaped its diversification. An alignment of the sequences of the members of this family reveals that they share only 13 seed sequences and that these seeds might have evolved by seed shifting and point mutation (Figure 1).

**Figure 2:**
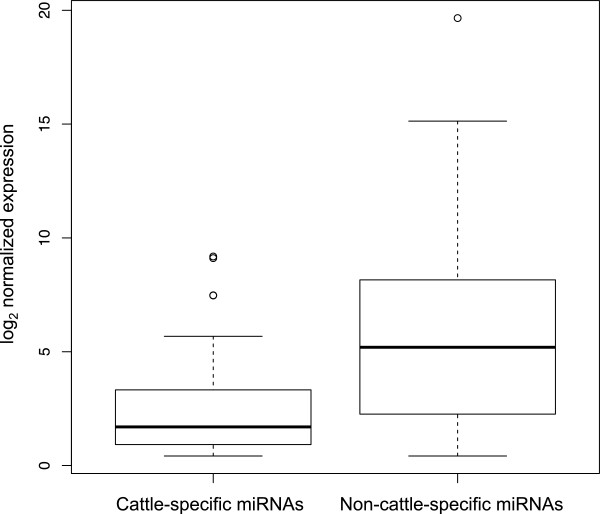
**Expression of cattle-specific versus non-cattle-specific miRNAs.** Box plots summarizing the expression levels of cattle-specific miRNAs and non-cattle-specific miRNAs in cattle blood. The y-axis represents the log2-transformed expression in counts per million mapped reads per sample (cpm).

Based on the numbers of reads obtained for each miRNA, most of the 71 cattle-specific miRNAs are expressed at a low level relative to the non-cattle-specific miRNAs, at least in blood (Figure 2). Of these 71, we consider 33 miRNAs with read count greater than 50 (corresponding to cpm above 1.5) as relatively highly expressed cattle-specific miRNAs. These 33 miRNAs included 14 members of the miR-2284 family of which bta-miR-2284x had the highest expression.

### Target sites of cattle-specific miRNAs show accelerated sequence evolution

**Figure 3:**
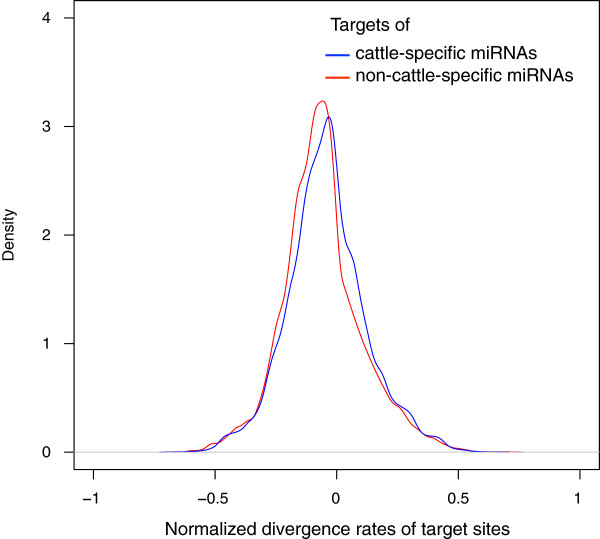
**The distribution of normalized divergence rates of miRNA target sites.** Density plots of the normalized target site divergence rates between cattle and human for cattle-specific (blue line) and non-cattle-specific miRNAs (red line). The targets of the cattle-specific miRNAs show a greater normalized divergence rate than the targets of the non-cattle-specific miRNAs (p = 2.2e-16, Kolmogorov-Smirnov test).

To better understand how target sites have evolved with the emergence of cattle-specific miRNAs, we first evaluated whether the cattle-specific miRNAs have a greater proportion of targets that are also cattle-specific. We used the predicted target sites of known and novel miRNAs from TargetScan (see Methods) and determined their positions on the 23-way UTR alignments available in TargetScan. Indeed a significantly higher (p < 0.0001, chi-square test) proportion of target sites were found to be specific to cattle (60%) for cattle-specific miRNAs compared to that (49%) for non-cattle-specific miRNAs (Table 3). Next, we compared the distribution of normalized divergence rates (defined as the divergence rate of the target site minus the divergence rate of the flanking regions) of target sites for cattle-specific versus non-cattle-specific miRNAs. The distribution for cattle-specific miRNAs displayed a shift towards the right indicating a significantly higher divergence rate (p < 2.2e-16, Kolmogorov-Smirnov test) and thus more rapid sequence evolution for target sites of cattle-specific miRNAs relative to those of non-cattle-specific miRNAs (Figure 3). The same trends were obtained when the context + score cutoff for predicting miRNA targets was relaxed (context + score cutoff = 90) or made more stringent (context + score cutoff = 99) indicating that the results were not dependent on the choice of context + score cutoffs. Thus we demonstrate that the emergence of cattle-specific miRNAs was accompanied by accelerated sequence evolution of their target sites.

**Table 3:** Targets of cattle-specific and non-cattle-specific miRNAs

**miRNA type**	**Predicted target type**		
	**Cattle-specific**	**Conserved**	**Total**
Cattle-specific	6937 (60%)	4535	11472
Non-cattle-specific	13425 (49%)	13969	27394

The predicted targets information is available from TargetScan for only the major miRNA sequences (not for the minor (star) sequences) i.e. 65 of the 71 cattle-specific and 183 of the 190 non-cattle-specific miRNAs.

### Decreased expression of the target genes of cattle-specific miRNAs

**Figure 4:**
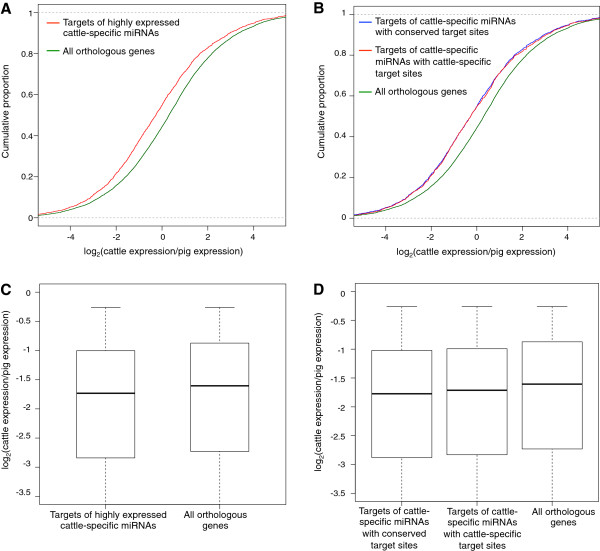
**Comparison of target mRNA expression between cattle and pigs.** Cumulative distribution function (CDF) plots and boxplots of log2-transformed gene expression (cpm) ratios. Each ratio is calculated as the expression of a bovine mRNA divided by the expression of its porcine orthologue. **(A)** The CDFs for targets of highly expressed cattle-specific miRNAs (n = 1699) and all orthologous genes (n = 8680) are significantly different (p = 1.014e-15) by the Kolmogorov-Smirnov test. Genes that are targeted by both conserved and cattle-specific miRNAs were excluded from this analysis. **(B)** The CDFs for targets of highly expressed cattle-specific miRNAs with conserved target sites (n = 757) and all orthologous genes (n = 8680) are significantly different (p = 1.118e-11) by the Kolmogorov-Smirnov test, as are the CDFs for targets of highly expressed cattle-specific miRNAs with cattle-specific target sites (n = 1096) and all orthologous genes (p = 1.124e-10). Target genes containing both conserved and cattle-specific target sites were excluded. **(C)** Boxplot of expression ratios for targets of the highly expressed cattle-specific miRNAs (median logFC = −1.73) and all orthologous genes (median logFC = −1.60). **(D)** Boxplot of expression ratios for target genes of highly expressed cattle-specific miRNAs having conserved target sites (median logFC = −1.77), having cattle-specific target sites (median logFC = −1.77), and target genes of conserved miRNAs (median logFC = −1.60).

Although our analyses suggest that there have been substantial additions to the miRNA and miRNA target repertoire during the evolution of cattle relative to pigs and other mammals, an important question remaining in this study is whether cattle-specific miRNAs contribute to gene expression divergence. To test this we measured mRNA expression in the whole blood of seven cattle and three pigs using RNA-seq. We used the distribution of expression ratios of all orthologous genes between cattle and pig as the genome-wide background (n = 8680). The target genes of 33 highly expressed cattle-specific miRNAs were indeed expressed at lower levels in cattle relative to their pig orthologues, as indicated by a leftward shift in the cumulative distribution of the mRNA expression ratio for the highly expressed cattle-specific miRNAs relative to the genome-wide background (p = 1.014e-15, Kolmogorov-Smirnov test) (Figure 4A). Next we tested whether cattle-specific miRNAs tend to decrease target gene expression by binding to cattle-specific or conserved target sites. Targets of cattle-specific miRNAs with conserved target sites and those with cattle-specific target sites showed reduced expression in cattle relative to the genome-wide background (p = 1.118e-11 and 1.124e-10 respectively, Kolmogorov-Smirnov test) (Figure 4B). The distributions for the target genes of cattle-specific miRNAs with conserved target sites (blue line in Figure 4B) were not significantly different (p = 0.4, Kolmogorov-Smirnov test) from those with cattle-specific target sites (red line in Figure 4B). We observe a similar pattern of expression divergence when using an expression data set from human blood (GEO Series accession number GSE33701) [25] as the one we generated from pigs (Additional file 5).

We next looked at the magnitude of expression reduction of targets of highly expressed miRNAs compared to genome-wide background. We only looked at the genes with fold change between pig and cattle greater than 1.2. The targets of the highly expressed cattle-specific miRNAs (median logFC = −1.73) showed significantly more reduction (p = 0.0065, Mann–Whitney U test) than the genome-wide background (median logFC = −1.60) (Figure 4C). Thus the target genes of highly expressed cattle-specific miRNAs showed 8% ((1.73-1.60)/1.60) greater expression reduction compared to the genome-wide background. Dividing the targets of the highly expressed cattle-specific miRNAs into those having cattle-specific or conserved target sites, we found that the degree of reduction in expression for both types (median logFC = −1.71 and −1.77 respectively) was significantly greater (p = 0.052 and 0.012 respectively, Mann–Whitney U test) than the genome-wide background (Figure 4D). The reduction in expression of target genes with conserved target sites of the highly expressed cattle-specific miRNAs was not significantly different from the target genes with cattle-specific targets (p > 0.05, Mann–Whitney U test).

### Functional enrichment of target genes of cattle-specific miRNAs

In order to assess the biological effects of the 33 highly expressed cattle-specific miRNAs, we looked for enriched biological pathways among the genes they target. We examined KEGG pathway enrichment for targets expressed in blood (n = 1708) and for expressed targets with reduced expression in cattle compared to pigs (n = 856) and human (n = 754). Because miRNAs expressed in blood can target genes in other tissues [26], we also looked at all predicted targets (n = 3255) irrespective of their expression in blood. Although most of the KEGG pathways found to be enriched (p < 0.05 and gene count > =3) did not show an obvious relationship to cattle-specific functions (Additional file 6), the insulin signalling pathway, which is known to contribute to metabolic differences between ruminants and non-ruminants, is enriched in targets expressed in blood (p = 0.044) and in expressed targets with reduced expression in cattle compared to human (p = 0.032) but not pig (p = 0.165). The insulin signalling pathway showed a p-value of 0.059 when considering all predicted targets of the 33 highly expressed cattle-specific miRNAs. Notably, cattle-specific miRNAs may target some of the key annotated insulin signalling pathway genes, including AKT3, CBLB, FOXO1 and PIK3R5 (all show reduced expression in cattle compared to human and pig).

## Discussion

In this study, 23% of the miRNAs identified from cattle whole blood are found to have no homologs in 57 other vertebrate species examined. Based on this set of cattle-specific miRNAs, we can provide an estimate of the net gain rate of new miRNAs during cattle evolution. Given the estimated 64.5 Myr (million years) divergence time between cattle and pig [27] and the 71 cattle-specific miRNAs we identified, the net gain rate of miRNAs expressed in blood is estimated as 1.1 miRNAs per Myr. This is about twice the rate of that observed in pigs (0.6 miRNAs per Myr). One of the most interesting cases is the bta-mir-2284 family, which has 24 members. Why does the cattle genome maintain so many members in this family? The abundant miRNA seeds generated by seed shifting and point mutation in this family indicate that the emergence of novel miRNAs may have led to adaptive functional diversification. However, the number of unique seeds is much less than the number of paralogues and many miRNA members share the same seed sequence, suggesting that dosage effect might be also important for the function of mir-2284 family.

It has long been hypothesized that gene expression changes are one of the main underlying causes of phenotypic differences between species [1,2]. While divergence in cis-acting elements and trans-acting proteins has been shown to underlie evolutionary divergence [3,4], relatively little is known about the role of miRNAs in shaping gene expression divergence. Here we showed that both the proportion of genes with decreased expression and the degree of expression reduction (relative to their pig and human orthologues) are higher for targets of cattle-specific miRNAs compared to genome-wide background. The target genes of cattle-specific miRNAs might have been under selection for decreased expression, which has been achieved by several means, one of them being cattle-specific miRNAs. However, the fact that the target genes of highly expressed cattle-specific miRNAs show a greater reduction in expression than those of the cattle-specific miRNAs expressed at low levels (Additional file 7) further implicates miRNAs as the major player in shaping the expression patterns of these genes, as opposed to other factors. Functionally, cattle-specific miRNAs might be associated with the insulin signalling pathway, and thus potentially have a role in the evolution of insulin resistance in ruminants. It would be worthwhile to analyse how species-specific miRNAs modulate target gene expression divergence across other model animal species for species-specific functions.

In this study, we found more target genes with cattle-specific target sites for cattle-specific miRNAs than for non-cattle-specific miRNAs and we observed accelerated sequence evolution of target sites of cattle-specific miRNAs. This accelerated evolution suggests that selection might have favoured the formation of new target sites. Previous studies have primarily focused on conserved target sites but our findings suggest that the non-conserved targets may represent novel mechanisms of genetic regulation that can contribute to species-specific phenotype. Based on target gene expression analyses, we showed that cattle-specific miRNAs have effects on targets genes of both types: those with conserved targets sites and those with cattle-specific target sites. Thus these miRNAs may fine tune pre-existing regulatory mechanisms as well as contribute to the formation of new regulatory mechanisms.

## Conclusions

We provide evidence of rapid miRNA-mediated regulatory evolution in the ruminant lineage. Cattle-specific miRNAs play an important role in shaping gene expression divergence between ruminant and non-ruminant species, by influencing the expression of target genes with either conserved or cattle-specific target sites. One interesting potential role for these miRNAs is to increase insulin resistance in ruminants by targeting insulin signalling.

## Methods

### Sample collection and RNA preparation

Three cattle and three pig blood samples were used for miRNA sequencing. One pooled sample from seven cattle blood samples and three separate pig blood samples were used for mRNA sequencing. Peripheral whole blood (approximately 2.5 mL) was collected from the jugular vein of cattle and pigs, into PAXgene Blood RNA tubes (BD, Cat. No. 762165) and processed according to the manufacturer’s instructions. Total RNA was extracted from 4.5 – 9.0 ml of solution from the PAXgene Blood RNA tubes using the PreAnalytiX kit (Qiagen, Cat. No. 763134). The quality and quantity of the RNA were determined using Agilent 2100 Bioanalyzer (Agilent Technologies, Santa Clara, CA) and Qubit 2.0 Fluorometer (Invitrogen, Carlsbad, CA). The animal study was approved by the Animal Care and Use Committee of the University of Alberta (Guan 019).

### Library construction and Illumina sequencing

Total RNA (1.5 μg for each sample) was used to construct miRNA and mRNA libraries using the TruSeq Small RNA and mRNA Sample Preparation Kit (Illumina, San Diego, CA) according to the manufacturer’s instructions. PCR amplification was performed for 12 cycles. Library quality for miRNA and mRNA libraries was determined using the High Sensitivity DNA Chip and an Agilent 2100 Bioanalyzer (Agilent Technologies). qRT-PCR was then performed for library quantification using the StepOne^TM^ Real-Time PCR System (Applied Biosystems, Carlsbad, CA) with the KAPA SYBR ® Fast ABI Prism qPCR kit (Kapa Biosystems, Woburn, MA).

The individual libraries were adjusted to 2 nM concentrations and pooled before denaturation and dilution according to Illumina’s instructions. The diluted libraries (8 – 10 pM) were loaded on a cBot (Illumina) for cluster generation using the TruSeq™ SR Cluster Kit v3 (Illumina). Sequencing was performed on the HiScan SQ system (Illumina) using the TruSeq™ SBS Kit v3 (50 cycels, Illumina). Real-time analysis and base calling was performed using the HiSeq Control Software version 1.4.8 (Illumina).

### Identification and quantification of known and novel miRNAs

Sequence reads with base quality scores were produced by the Illumina sequencer. Reads flagged as low-quality by CASAVA 1.8 were removed. After trimming the 3’ adaptor sequence, all sequences ranging in length from 18–26 nt were recorded in a non-redundant file along with copy number. Further analyses were performed using miRDeep2 [22,28] and custom Perl scripts. All tags were compared to the Rfam database of RNA families [29,30] to filter out non-miRNA sequences. To identify known miRNAs, the miRNA tags of the six samples were aligned against miRNA precursor sequences reported in the miRNA database ‘miRBase’ (release 18) [31-34] using the ‘quantifier.pl’ script (with the default parameters) within miRDeep2. Candidates for novel miRNAs were identified by miRNA precursor prediction within miRDeep2. For novel miRNA prediction, the following criteria as recommended by the authors of miRDeep2 [22] were chosen: miRDeep score cutoff of 5, which is estimated by miRDeep2 to yield a true positive rate > 90% and a signal-to-noise ratio >10. Each sample was processed separately and the results for each species were combined by genomic location. We considered only those miRNAs with read counts greater than five in all three samples as being expressed. The read counts per miRNA in each sample were normalized to counts per million mapped reads (cpm).

### Identification of miRNA homologs

A three-step procedure was used to find homologs of the known and novel cattle miRNAs identified. First, the mature sequences of the cattle miRNAs were aligned against the genomes of 57 vertebrate species with good quality assembled genomes available in the Ensembl database version 71 (http://www.ensembl.org/index.html) [35] using the short read aligner software Bowtie [36]. The hairpin (precursor) sequences of the cattle miRNAs with mature sequences that showed no more than two mismatches in the previous alignment step were then selected for a second round of alignments against the 57 vertebrate genomes. The nucleotide blast program (blastn) of NCBI’s ‘Basic Local Alignment Search Tool’ (BLAST) [37] was used to perform this alignment, with cutoffs for expected score set at below 0.1 and percent identity set at above 60% of the length of the hairpin sequence. Finally, the stabilities of the secondary structures of the predicted homologous hairpin sequences were tested based on their minimum free energy of folding (below −25 kcal/mol) using the RNA secondary structure prediction program RNAfold [38] within the Vienna RNA package (version 2.0) [39]. The cattle miRNAs with no homolog in any other species were classified as “cattle-specific” and those with a homolog in any other species were classified as “non-cattle-specific”.

### Sequence conservation of target seed sites

To evaluate the conservation of target seed sites of miRNAs across species, we used the predicted miRNA target sites with context + scores above 95^th^ percentile and determined their positions on the 23-way UTR alignments available in TargetScan (release 6.2, June 2012) [40-42]. The target predictions in TargetScan are made only for the major sequence and not the minor (star) sequence of the miRNAs. We used the Perl scripts provided on the TargetScan website (http://targetscan.org) for predicting and calculating the context scores for the targets of novel miRNAs.

For each cattle miRNA target seed site the aligned sequences from five other species available in the 23-way UTR alignments were examined: human, dog, mouse, rat and chicken (the pig genome was not available in this multi-species alignment). If the target site was not perfectly conserved with any of the five species considered (both nucleotide substitutions and indels in the other species were considered as divergence), then the target site was classified as cattle-specific.

We used the method described by Zheng *et al*. [43] to calculate the normalized divergence rates of target sites between cattle and human. The normalized divergence rate is defined as the divergence rate of the target site minus the divergence rate of flanking region. The divergence rate of the target site is defined as the number of nucleotide substitutions in the target sequence divided by its length (7-mer or 8-mer) and the divergence rate of the flanking regions is defined as the number of nucleotide substitutions in the upstream and downstream regions, divided by the corresponding flanking region lengths (84 and 96 respectively for flanking regions of 7-mer and 8-mer seed sequences).

### Comparison of expression levels of cattle mRNAs with their orthologues in pig and human

Sequence reads with base quality scores were produced by the Illumina sequencer. RNA-seq reads flagged as low-quality by CASAVA 1.8 were removed. Cattle and pig reads were aligned to the cattle (UMD 3.1) and pig (Sus 10.2) reference genome sequences respectively using Tophat 1.4.0 with default parameters [44]. The number of reads mapped to each gene was determined using htseq-count (v0.5.3.p3). The read counts per gene were normalized to cpm.

The pig and human orthologues of cattle mRNAs were determined using the homology mappings provided in the BioMart biological database (version 0.7) [45] (http://ensembl.org/biomart/martview/). A total of 8680 orthologous pairs between cattle and pig showed cpm > 0.5 in both species and were used for downstream analyses. Similarly, we identified 9442 orthologous pairs between cattle and human. The RNA-seq-determined expression levels (expressed in cpm) of the cattle mRNA targets were then compared to those of their porcine and human orthologues for various subsets of targets, using cumulative proportion plots and Kolmogorov-Smirnov and Mann–Whitney U statistical tests.

### Test for biological pathway enrichment among target genes of highly expressed cattle-specific miRNAs

The target genes (predicted by TargetScan with context + scores above 95^th^ percentile) of 33 highly expressed cattle-specific miRNAs were subjected to KEGG pathway [46] enrichment analysis using the ‘GOstats’ tool [47] within the Bioconductor package (version 2.12) of the R statistical programming language (version 3.0.1) [48]. Of the 390 KEGG terms from the ‘KEGG.db’ annotation map [49], we used 375 for the enrichment testing after excluding 15 cancer-related terms. Conversions between bovine Ensembl IDs, Entrez gene IDs and gene symbols, when needed, were done using the genome wide annotation map for bovine available from the Bioconductor package ‘org.Bt.eg.db’ [50].

## Availability of supporting data

The miRNA and mRNA sequence data sets described in this article are available from the NCBI Sequence Read Archive under accession ID SRP018740 at http://trace.ncbi.nlm.nih.gov/Traces/sra/?study=SRP018740.

## Competing interests

The authors declare that they have no competing interests.

## Authors’ contributions

HB and AK conceived study, carried out the analyses and wrote the manuscript. XS and YM performed the experiment. ASA contributed to sequencing data management. GSP involved in discussion and manuscript revision. PS and LLG conceived and supervised the study, and revised the manuscript. All authors revised the manuscript and approved the final version.

## Authors’ information

Co-first authors; Hua Bao and Arun Kommadath.

## Supplementary Material

Additional file 1 **Mapping statistics of miRNAs data.** Spreadsheet contains the numbers of reads sequenced and mapped from cattle and pig miRNA libraries. Click here for file

Additional file 2 **Known cattle and pig miRNAs identified by miRDeep2.** Spreadsheet contains the numbers of reads mapped to known miRNAs. Click here for file

Additional file 3 **Novel cattle and pig miRNAs predicted by miRDeep2.** Spreadsheet contains the position and sequence of novel miRNAs predicted by miRDeep2. Click here for file

Additional file 4 **Cattle-specific miRNAs identified in this work.** Spreadsheet contains the known and novel cattle-specific miRNAs based on conservation analysis. Click here for file

Additional file 5 **Comparison of target mRNA expression between cattle and human.** PDF file contains the cumulative distribution function (CDF) plots of log2-transformed gene expression (cpm) ratios. Click here for file

Additional file 6 **Enriched pathway for targets of cattle-specific miRNAs.** Spreadsheet contains the KEGG pathway enriched among predicted targets of the highly expressed cattle-specific miRNAs. Click here for file

Additional file 7 **Comparison of mRNA expression for targets of highly versus lowly expressed cattle-specific miRNAs.** PDF file contains the cumulative distribution function (CDF) plots of log2-transformed gene expression (cpm) ratios. Click here for file
