# Supplementary material for: Expansion of ruminant-specific microRNAs shapes target gene expression divergence between ruminant and non-ruminant species
Source: BMC Genomics. 2013 Sep 10;14:609. doi: 10.1186/1471-2164-14-609 (PMC3847189; doi:10.1186/1471-2164-14-609)
Supplement: Additional file 5 — Comparison of target mRNA expression between cattle and human. PDF file contains the cumulative distribution function (CDF) plots of log2-transformed gene expression (cpm) ratios. [file 1471-2164-14-609-S5.pdf]

Additional file 5- Comparison of target mRNA expression between cattle and human

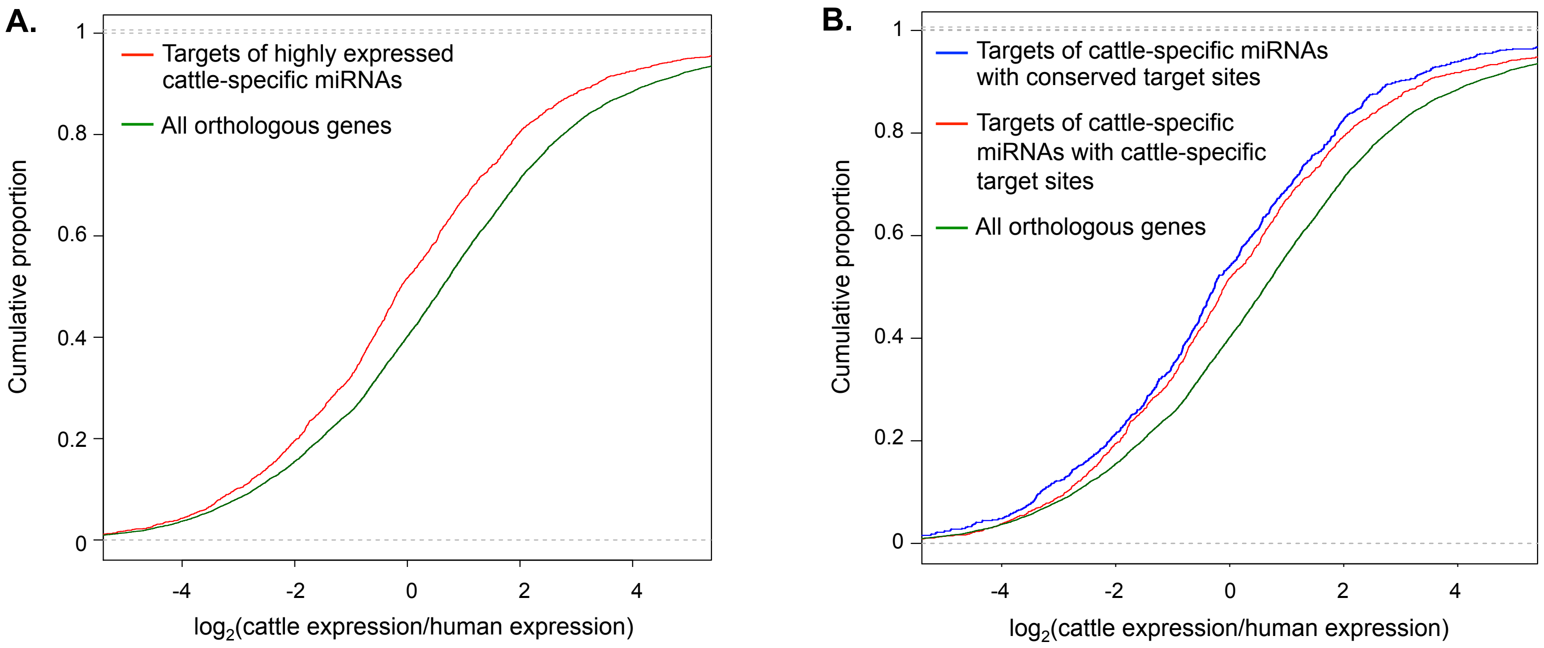

Cumulative distribution function (CDF) plots of log2-transformed gene expression (cpm) ratios. Each ratio is calculated as the expression of a bovine mRNA divided by the expression of its human orthologue. (A) The CDF for targets of highly expressed cattle-specific miRNAs (n= 1829) and all orthologous genes (n= 9442) is significantly different ($p < 2.2e-16$) by the Kolmogorov-Smirnov test. Genes that are targeted by both conserved and cattle-specific miRNAs were excluded from this analysis. (B) The CDFs for targets of highly expressed cattle-specific miRNAs with conserved target sites (n= 830) and all orthologous genes (n=9442) are significantly different ($p = 8.554e-16$) by the Kolmogorov-Smirnov test, as are the CDFs for targets of highly expressed cattle-specific miRNAs with cattle-specific target sites (n= 1161) and all orthologous genes ($p = 2.821e-13$). However, the CDF for targets of highly expressed cattle-specific miRNAs with conserved target sites is not significantly different from the CDF for targets of highly expressed cattle-specific miRNAs with cattle-specific target sites ($p=0.25$).
