# Supplementary material for: Expansion of ruminant-specific microRNAs shapes target gene expression divergence between ruminant and non-ruminant species
Source: BMC Genomics. 2013 Sep 10;14:609. doi: 10.1186/1471-2164-14-609 (PMC3847189; doi:10.1186/1471-2164-14-609)
Supplement: Additional file 7 — Comparison of mRNA expression for targets of highly versus lowly expressed cattle-specific miRNAs. PDF file contains the cumulative distribution function (CDF) plots of log2-transformed gene expression (cpm) ratios. [file 1471-2164-14-609-S7.pdf]

Additional file 7- Comparison of mRNA expression ratios for targets of highly versus lowly expressed cattle-specific miRNAs

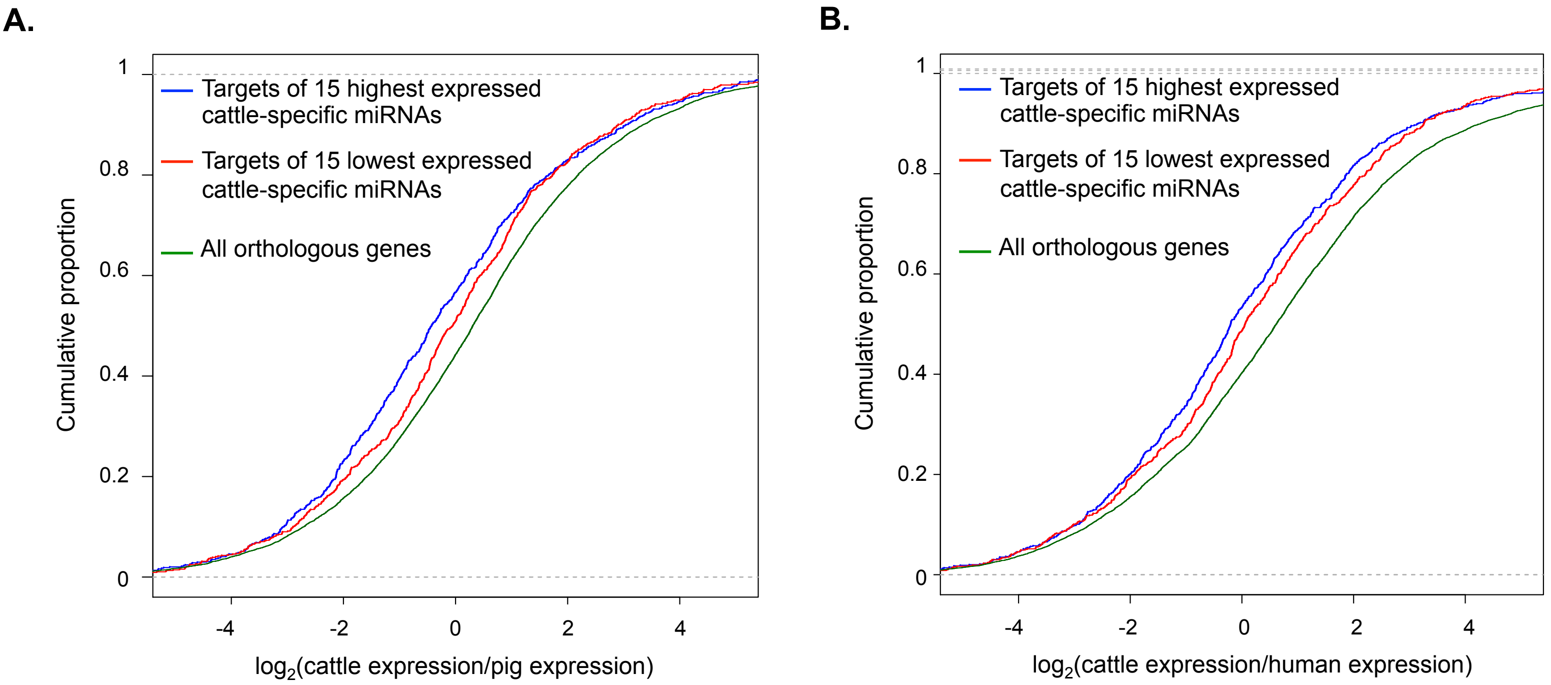

Cumulative distribution function (CDF) plots of log2-transformed gene expression (cpm) ratios. Each ratio is calculated as the expression of a bovine mRNA divided by the expression of its pig (A) and human (B) orthologue. (A). The CDFs for targets of the 15 highest expressed cattle-specific miRNAs (n=742) and targets of 15 lowest expressed cattle-specific miRNAs (n=766) are significantly different (p = 0.0017) by the Kolmogorov-Smirnov test when considering pig orthologues. (B). Similarly, the difference between the CDFs for targets of 15 highest expressed cattle-specific miRNAs (n=796) and targets of 15 lowest expressed cattle-specific miRNAs (n=835) are significant (p=0.016) when considering human orthologues.
